# Prevalence and outcome of injury in patients visiting the emergency Department of Yirgalem General Hospital, Southern Ethiopia

**DOI:** 10.1186/s12873-018-0165-6

**Published:** 2018-05-22

**Authors:** Abel Negussie, Andarge Getie, Elias Manaye, Tamrat Tekle

**Affiliations:** 1 Department of Social and Population Health, Yirgalem Hospital Medical College, Yirgalem, Ethiopia; 2 Administration for Refugee and Returnee Affairs (ARRA), Addis Ababa, Ethiopia; 3 Department of Internal Medicine, Wollo University, Dessie, Ethiopia; 4 Dawro General Hospital, SNNPR, Dawro, Ethiopia

**Keywords:** Injury, Prevalence, Outcome, Facility-based, Southern Ethiopia

## Abstract

**Background:**

Traumatic injuries continue to be an important cause of morbidity and mortality in the developing world. Despite the high burden of injury in Ethiopia, the occurrence and health impact have not received due attention. The aim of the study was to assess the prevalence and outcome of injury among patients visiting the Emergency Department (ED) of Yirgalem General Hospital, southern Ethiopia.

**Methods:**

A facility-based prospective cross sectional study was conducted from March, 27 – April, 30/2017. The final calculated sample size was 353 and all eligible trauma patients who visited the ED of Yirgalem General Hospital during the study period were included in the study. Data was collected using a checklist which was adapted from the WHO injury surveillance guideline. The data were entered and analyzed using SPSS version 19.

**Results:**

A total of 346 patients, who visited the ED during the study period, participated in the study and of them, 171 (49.4%) were injury cases. Unintentional injuries accounted 123 (71.9%) of the total injuries and the age group ≤24 years (48.2%) was the most commonly affected age group. More than half (51.4%) of unintentional injury cases were due to Road Traffic Injuries (RTIs) and 48 (28%) of the cases were attributed to interpersonal violence (assault). The majority of patients, 97 (56.7%), had a minor or superficial injury (like bruises and minor cuts), 44 (25.7%) had a moderate injury and 16 (9.3%) had severe type of injury requiring intensive medical/surgical management; and RTIs accounted for 11 (68%) of all severe injuries.

**Conclusion:**

The prevalence of injury was considerably high in Yirgalem General Hospital. Road Traffic Injuries (RTIs) accounted for the majority of severe injury cases; therefore, appropriate prevention strategies should be strengthened and implemented against RTIs. We also suggest that children and young adults should be educated in schools and work environments to prevent injuries/accidents.

## Background

Injuries continue to be an important cause of morbidity and mortality in the developed and developing world. According to WHO Global Burden of Disease (GBD) estimates, in 2008, 5.1 million people died worldwide every year as a result of injuries and violence. This account for around 9% of the world’s deaths and the majority of injury related deaths are unintentional or “accidental” with road traffic crashes, falls and drowning ranking among the main causes [1]. In the past few decades, due to urbanization, industrialization, rapid motorization, and unsafe driving, the risk of accidents have increased, contributing a large burden of mortality and morbidity, especially in developing countries [2]. Road Traffic Injuries (RTIs) are among the leading causes of death and lifelong disability globally and according to the 2015 WHO global status report on road safety, about 1.25 million people die annually on the world’s roads, with 20–50 million sustaining non-fatal injuries [3].

Traumatic injuries represent a significant and growing disease burden in the developing world, and now it is one of the leading causes of death in economically active adults in many low and middle income countries (LMICs) [4]. LMICs represent more than 90% of global road traffic deaths and RTI death rates are highest in the African region. In developing countries, injury ranks third as a major cause of death and permanent disability among the adult population next to tuberculosis and HIV/AIDS [5]. The main reason of this burden of injuries is due to lack of organized efforts to reduce its occurrence; and the cost-effectiveness of injury prevention and emergency treatment in these resource limited settings is not yet well understood as the development of emergency care systems is in its nascence [6].

In Ethiopia, like that of other developing countries, injuries are common and the 2008 national report on road safety indicated that nearly 19,000 road traffic accidents occurred per year, claiming over 2500 lives and property worth of US$ 56 million [7]. Even though some previous studies done in other regions of the country indicated high burden of injury, the occurrence and increasing public health importance haven’t received due attention and a comprehensive injury data still remains limited [8–10]. It is also important to provide local data on the rate of injuries which might serve as a baseline data for local authorities and health care planners to implement appropriate injury prevention strategies. The aim of this study was to describe the magnitude and outcome of injuries requiring ED visit in Yirgalem General Hospital, southern Ethiopia.

## Methods

### Study design and setting

A hospital-based prospective cross sectional study was conducted from March, 27 – April, 30/2017 at ED of Yirgalem General Hospital. Yirgalem General Hospital is found in Yirgalem town and it is one of the General Hospitals in the South, Nations, Nationalities and Peoples Region (SNNPR). The hospital was established in January 1966 and it serves about 4.2 million people. The hospital had four main departments (Medical, Surgical, Pediatrics and Gynecology/Obstetric wards), three special care units (Medical Intensive Care Unit, Neonatal Intensive Care Unit and Surgical recovery Room) and five clinics (Eye, Anti-retro viral Treatment, Dental, TB and MDR-TB clinics).

In the Ethiopian health care system, hospitals are structured as Primary, General and Specialized hospitals. Primary hospital (to cover 60,000–100,000 people) is established at district or primary care level; and the coverage of General and Specialized hospitals extends to larger portions of the population providing services to 1–1.5 million and 3.5–5 million people, respectively. Though the study was done in Yirgalem General Hospital, it may give the overall picture of the burden of injury at EDs of similar hospitals found in the country.

### Study participants

All eligible patients who visited the ED of Yirgalem General Hospital during the study period (March, 27 – April, 30/2017) were included in the study. Patients who died before arrival with unknown cause were excluded from the study. The required sample size to estimate the prevalence of injury was calculated using *P* = 0.32 [8], confidence interval = 95% and margin of error (d) = 5%. As a result of adding non-response rate of 5% to the initially estimated sample size, the final sample size became 353.

### Data collection procedure

Data was collected using a checklist which was adapted from the WHO injury surveillance guideline and comprised of items regarding the socio-demographic characteristics; and the mechanism, severity and outcome/type of injury cases. Data was collected by three clinical nurses who took a 2 days training on detail contents of the checklist. The filled checklists were checked for completeness and proper filling by the investigators to assure data quality.

### Data processing and analysis

The data were entered and analyzed using SPSS version 19. Frequency distribution and percentage calculations were made to describe the socio-demographic characteristics of study participants; and also the mechanism, severity and outcome of injury cases were examined using measures of descriptive statistics.

### Operational and standard definitions

- ***Injury:*** *Any instance of physical damage to the body or body part.*
- ***Intentional injury:*** *Categorized as interpersonal violence (including violence against intimate partner), collective violence (including war) and self-directed violence (suicide).*
- ***Psychoactive substance:*** *Is defined as psychoactive ingredients that alter mood, cognition and behaviour.*
- ***Outcome of the injury:*** *the intent of the injury that results on the victim like fracture, dislocation, laceration and abrasion.*
- ***Severity of injury***
***No injury:*** *Patient who had no accessible injury and no need of any intervention.*
  ***Minor injury:*** *Patient who had minor injury or superficial injury (*e.g. *Bruises, minor cut) requiring cleaning of the area.*
***Moderate injury:*** *Patient who had moderate injuries requiring some sort of skilled treatment such as fracture stabilization and suturing of wounds.*
  ***Severe injury:*** *Patient who had severe injuries requiring intensive medical/surgical management (*e.g. *internal haemorrhage, moderate/severe head injuries).*
- ***Unintentional injury:*** *Comprises road traffic injuries, fires, falls and drowning.*

## Results

Out of the total 353 patients who visited the ED during the study period, 346 of them agreed to participate in the study with a response rate of 98%; and of them, 171 (49.4%) were injury cases. Most study participants were males (67.3%), under the age group of 25 (48.2%), Sidama (77.2%) by ethnicity and Protestant (59.5%) by religion. Regarding the educational status of the participants, almost half 166 (48%) of them had attended primary education. Furthermore, students and farmers accounted 97 (28%) and 81 (23.4%) of the total ED cases, respectively (Table 1).

**Table 1:** Socio-demographic characteristics of study participants visiting the Emergency Department of Yirgalem General Hospital, southern Ethiopia, from March 27 – April 30/ 2017; (*n* = 346)

Variable	Categories	Frequency	Percent
Age	≤24	167	48.2
	25–59	158	45.6
	≥60	21	6.1
Sex	Male	233	67.3
	Female	113	32.7
Ethnicity	Sidama	267	77.2
	Amhara	42	12.1
	Oromo	30	8.7
	Others	7	2
Religion	Protestant	206	59.5
	Orthodox	112	32.4
	Muslim	28	8.1
Marital Status	Married	187	54
	Single	156	54.1
	Others	3	0.9
Residence	Urban	159	46
	Rural	187	54
Monthly Income (In Ethiopian Birr per month)	< 660	138	39.9
	660–1600	106	30.6
	> 1600	102	29.5
Educational level	Illiterate	87	25.1
	Primary education	166	48
	Secondary education	64	18.5
	Higher education	29	8.4
Occupational status	Student	97	28
	Farmer	81	23.4
	Unemployed	62	17.9
	Civil servant	34	9.8
	Trader of any kind	25	7.2
	Daily laborer	19	5.4
	Driver	18	5.2
	Construction worker	10	2.9

**Fig. 1:**
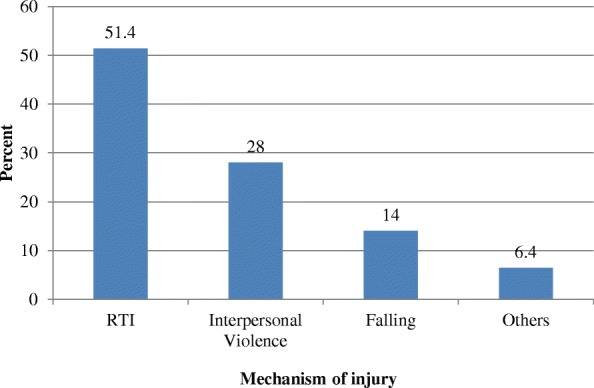
Mechanism of injury in patients visiting the Emergency Department of Yirgalem General Hospital, southern Ethiopia, from March 27 – April 30/2017; N = 171; blue square Mechanism of injury

The prevalence of injury was found to be 49.4% and unintentional injuries accounted 123 (71.9%) of the total injuries. More than half of the cases, 88 (51.4%), were due to RTIs followed by interpersonal violence (assault), 48 (28%), and falling accident, 24 (14%). Other less common causes of injury were burning, choking and injuries during use of industrial machineries (Fig. 1). Most assaults, 9 (18.3%), had used club/stick followed by hit/attack by a person, 7 (14.2%).

Of the total injury cases, 57 (33.3%), 42 (24.6%), 16 (9.3%) and 15 (8.7%) of the injured patients had a bruise/superficial injury, cuts/bites/open wound, fracture and concussion, respectively (Table 2). The majority of patients, 97 (56.7%), had a minor or superficial injury (like bruises and minor cuts), 44 (25.7%) had a moderate injury and 16 (9.3%) had severe type of injury requiring intensive medical/surgical management. Most importantly, RTIs alone accounted for 11 (68%) of all severe injuries. Our study found that 53 (31%) of the injured patients had a history of alcohol consumption before 6 Hrs. of the trauma. Of them, 26 (49%) had encountered assault and 10 (5.8%) had used psychoactive substance in addition to alcohol.

**Table 2:** Type of injury in patients visiting the Emergency Department of Yirgalem General Hospital, southern Ethiopia, from March 27 – April 30/2017, (*n* = 171)

Type of injury	Frequency	Percent (%)
Bruise	57	33.3
Cut/open wound	42	24.6
Strain	18	10.5
Fracture	16	9.3
Concussion	15	8.7
Dislocation	8	4.6
Organ system	5	2.9
Concussion and open wound	6	3.5
Concussion and organ system	3	1.7
Others	10	5.8

**Fig. 2:**
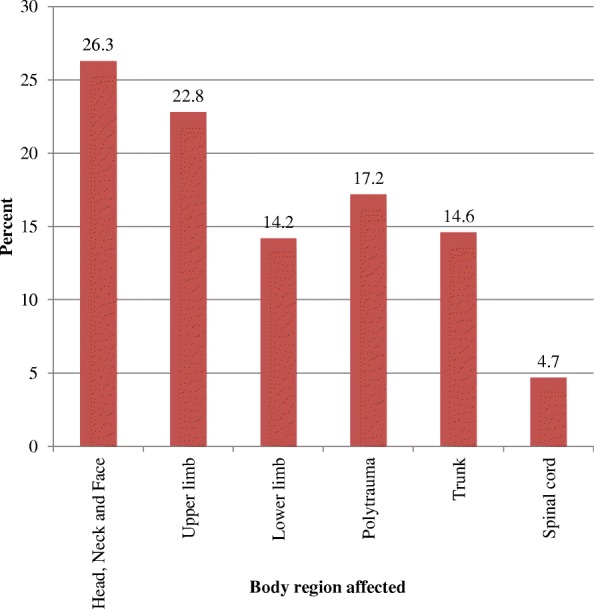
Body part affected by the injury in patients visiting the Emergency Department of Yirgalem General Hospital, southern Ethiopia, from March 27–April 30/2017; *N* = 171; red square Body region affected

Most frequent affected body parts were head, neck and face – 45 (26.3%). Upper extremity, 39 (22.8%), and trunk injuries, 25 (14.6%), were also common (Fig. 2). The majority of the cases, 135 (78.9%), were treated as outpatients, 27 (15.7%) were admitted and 9 (5.2%) were referred to other hospitals for further investigation and management. All outpatient and inpatient cases, 162 (100%), were discharged with improvement and follow-up.

## Discussion

The study found that half of all emergency visits were due to traumatic injuries. This result is higher when compared to other similar study findings [8, 11], but is relatively comparable to the report of a study done in Amhara Regional State Referral Hospitals, Ethiopia (55.6%) [9]. This difference in the magnitude of injury cases may be due to differences in study time and setting; or differences in the level and number of facilities studied. The most common cause of injury was found to be RTIs which is similar to the findings of other researches [4, 9, 11]. Furthermore, a greater number of injury victims were under the age of 25 compared to those aged 25–59 or to those aged 60 years and older, which signifies the possible economic impact of the problem as the productive age group of the society is being affected. The possible explanation for this may be is that, this age group is in active working years of life and it is time of practicing independent life out of parental supervision. This finding is also in line with other previous studies [5, 10].

Our study also indicated that RTI was the most common cause of injury contributing to more than half of the total unintentional injuries. This result is higher than the finding of a study done at Regional referral hospitals in Amhara region, Ethiopia. This may be due to lack of well-established road for pedestrians, as the primary victims of RTIs were the pedestrians, and no separate pathways for domestic animals in the study area, or due to the difference in study time and setting [9].

We found that minor or superficial injuries were the leading injury outcomes and unlike our finding, a study done in Jimma, Ethiopia, showed that most of the cases were with moderate injury [9]. Moreover, a study done at Tikur Anbessa Specialized Referral Hospital, Addis Ababa, reported that fractures were the primary outcome of injuries [8]. This discrepancy may be attributed to a difference in status of the hospitals in the health care delivery system. Most importantly, RTIs alone accounted more than half of the severe injuries, in our study. In addition, the majority of injury patients were treated and discharged as outpatients, which is comparable to other studies done elsewhere [10, 11].

### Limitations of the study

Even though the study indicated the prevalence and outcome of injury among emergency department patients, it is a descriptive facility based study and lacks generalizability to the total target population. Additionally, patients who had visited the emergency department during a specific period were considered and a possible selection bias may present.

## Conclusions

Our study found that the prevalence of injury was considerably high in Yirgalem General Hospital, and the age group of ≤24 years was the most affected age group. RTIs accounted half of all severe injury cases; therefore, appropriate prevention strategies should be strengthened and implemented against RTIs. We also suggest that children and young adults should be educated in schools and work environments to prevent injuries/accidents as they are the most affected group. Furthermore, future studies which focus on identifying determinant factors of RTIs need to be conducted.

## Abbreviations

ED: Emergency Department
LMICs: Low and middle income countries
RTI: Road traffic injury
WHO: World Health organization
